# Dataset on flue gas composition during combustion in the fluidised bed reactor. Glycerol combustion

**DOI:** 10.1016/j.dib.2020.105418

**Published:** 2020-03-12

**Authors:** Witold Żukowski, Gabriela Berkowicz

**Affiliations:** Faculty of Chemical Engineering and Technology, Cracow University of Technology, ul. Warszawska 24, 31-155 Cracow, Poland

**Keywords:** Flue gases composition, Glycerol combustion, Fluidised bed, Cenospheres, Fe_2_o_3_-cenospheres

## Abstract

The dataset presented in this article is the supplementary data for the research article by Żukowski and Berkowicz (doi:10.1016/j.combustflame.2019.05.024) [1], in which for the first time the inert and catalytic cenospheres were used as the fluidised bed material, giving the possibility to burn liquids inside the fluidised bed without the need for using specialised dosing systems. The instantaneous concentrations of the gaseous products during the combustion of glycerol samples were detected using Fourier Transform Infrared Spectroscopy (FTIR, Gasmet DX-4000). The accurate composition at the outlet of the reactor makes it possible to evaluate and verify new kinetic models of the glycerol combustion in the fluidised bed. It also will be helpful in creating new simplified models. The data presented here is essential for the evaluation of CFD combustion models which have to include accurate kinetic data.

**Specifications table**

**Table:** 

Subject	Chemical Engineering; Environmental Engineering
Specific subject area	Combustion processes, Fluidisation in the inert and catalytic bubbling bed
Type of data	Table
How data were acquired	The species and concentrations of the main gaseous products during glycerol combustion were obtained by using infrared absorption spectroscopy (FT-IR, DX-4000, Gasmet Technologies).
Data format	Raw
Parameters for data collection	A careful selection of the analysed compounds was made, and for each compound, a suitable wavenumber range was chosen to avoid overlapping with the spectra of other components. Selected FTIR wave number ranges are available as supplementary material in the related research article [1].
Description of data collection	Combustion of glycerol was carried out in a fluidised bed reactor. Two kinds of beds were used. The inert bed was made of cenospheres. The catalytic bed was built of cenospheres covered with Fe_2_O_3_. The beds were heated to 400–900 °C and then glycerol samples were dripped from the top of the reactor. Quantitative analyses of flue gas composition were performed by GASMET software. This software makes the deconvolution of the spectra of the gas mixture using the method of the least-squares. The concentrations of the main flue gas components were obtained as a result of deconvolution.
Data source location	Cracow University of Technology, Faculty of Chemical Engineering and Technology, Cracow, Poland
Data accessibility	Data are accessible with the article.
Related research article	Witold Żukowski, Gabriela Berkowicz, The combustion of liquids and low-density solids in a cenospheric fluidised bed, Combustion and Flame, doi:10.1016/j.combustflame.2019.05.024

## Value of the data

• The data provides detail information on the quality and quantity of the gaseous products during the combustion of glycerol in fluidised bed made of inert or catalytic cenospheres.
• These data made it possible to develop the new fluidised combustion models.
• The data indicates products which are present in the flue gases at given temperatures. They can be used to check already known models of fluidised bed combustion as well as to facilitate the selection of necessary simplifications and skeletons when creating new models.
• The data may be of interest to individual scientists as well as the largest groups involved in modelling (like The CRECK modelling Group from Politecnico di Milano [2], Lawrence Livermore National Laboratory [3], Combustion group from Berkeley University of California [4], The combustion research group from UC San Diego [5], Combustion Kinetics Laboratory from University of Southern California [6].
• Additionally, the data present evidence that Fe_2_O_3_ can be used as a catalyst for heterogeneous oxidation to reduce the formation of undesired NO_x_ via the prompt mechanism.

## 1 Data description

The modelling of combustion processes is the subject of many works. In the face of current climate problems, low-emission fluidised bed combustion should attract more attention. However, modelling the combustion process in a fluidised bed reactor is a complex task [7]. To obtain the correct modelling results, it is necessary to validate the kinetic parameters of the chemical reactions with a description of mass and heat exchange in the reaction environment. To validate a chemical process model with a specific environment, experimental data is required to provide as much information as possible about the composition of chemical products. In the case of a process carried out for a single portion of fuel introduced into the bed, the data on the dynamics of the chemical composition of exhaust gases over time is extremely important and it is showed here.

This work present composition of flue gases recorded by the FTIR analyser during the combustion of glycerol in a fluidised bed made of cenospheres. Glycerol is a waste liquid derived from the production of biodiesel [8]. It is estimated that its quantities will increase. There are attempts to model glycerol steam reforming or pyrolysis [9,10] because of the possibility of hydrogen production. Burning waste glycerol continuously in a fluidised bed is also a considered waste management option [11]. As there are no papers presenting well-validated glycerol combustion models, it is believed that sharing the composition of the exhaust gases from a real object during the combustion of fuel in a fluidised bed is particularly important for the further development of modelling of combustion processes.

This dataset contains 11 Tables. Table 1 presents process variables describing features of the fluidised bed reactor, bed material, and fluidising air. Tables 2–6 contain flue gas composition during glycerol combustion in the inert bed. Tables 7–11 contain flue gas composition during glycerol combustion in the catalytic fluidised bed.

**Table 1:** Parameters of fluidised bed reactor, bed material and fluidising medium.

Parameter, units	Magnitude	
Fluidised bed reactor		
Shape	tube	
Wall material	quartz	
Inner diameter, mm	96	
Height, mm	500	
Heating	electrical	
Sieve bottom	CrNi steel plate (1 mm thick), evenly distributed holes (diam. 0.6 mm; 6.25 cm^−2^)	
Types of fluidised bed		
Type	inert	catalytic
Material	cenospheres	cenospheres-Fe_2_O_3_
Shape	spherical	
Grain diameter, mm	0.25–0.30	
Mass, g	290	320
Density, g/cm^3^	845	930
Static height, mm	115	
Temperature, °C	400 - 900	
Minimum fluidisation velocity, cm/s	1.38 (at 400 °C)	1.52 (at 400 °C)
	1.24 (at 500 °C)	1.37 (at 500 °C)
	1.13 (at 600 °C)	1.25 (at 600 °C)
	1.04 (at 700 °C)	1.15 (at 700 °C)
Fluidising medium		
Physical state	Gas	
Composition	Air (79% N_2_, 21%O_2_)	
Temperature, °C	25	
Pressure, hPa	1013	
Flow Rate, L/min @STP	30

**Table 2:** Composition of flue gases during glycerol combustion in a fluidised bed of cenospheres at 400 °C.

**Table 3:** Composition of flue gases during glycerol combustion in a fluidised bed of cenospheres at 500 °C.

**Table 4:** Composition of flue gases during glycerol combustion in a fluidised bed of cenospheres at 600 °C.

**Table 5:** Composition of flue gases during glycerol combustion in a fluidised bed of cenospheres at 700 °C.

**Table 6:** Composition of flue gases during glycerol combustion in a fluidised bed of cenospheres at 800 and 900 °C.

**Table 7:** Composition of flue gases during glycerol combustion in a fluidised bed of Fe_2_O_3_-cenospheres at 400 °C.

**Table 8:** Composition of flue gases during glycerol combustion in a fluidised bed of Fe_2_O_3_-cenospheres at 500 °C.

**Table 9:** Composition of flue gases during glycerol combustion in a fluidised bed of Fe_2_O_3_-cenospheres at 600 °C.

**Table 10:** Composition of flue gases during glycerol combustion in a fluidised bed of Fe_2_O_3_-cenospheres at 700 °C.

**Table 11:** Composition of flue gases during glycerol combustion in a fluidised bed of Fe_2_O_3_-cenospheres at 800 and 900 °C.

## 2 Experimental design, materials and methods

The reactor was created from a quartz tube located on a chrome-nickel perforated plate. The parameters of the reactor and plate are summarised in Table 1. The table also includes the features of two fluidised bed materials (inert and catalytic cenospheres) that were placed in the reactor. The minimum fluidisation velocities were calculated from the Wen and Yu correlation [12]. Previous studies of fluidisation of cenospheres and Fe_2_O_3_-cenospheres (which were presented in the publication [1]) have shown that this correlation gives the results consistent with real ones. The beds were fluidised with 30 dm^3^/min of air (@ 25 °C, 1013 hPa) and were electrically heated to 400, 500, 600, 700, 800 and 900 °C. At each temperature, samples of glycerol were dripped into the bed from a height of about 500 mm where the temperature was ambient. The glycerol was purchased from the Chempur Company (Poland), and its density was 1.26 g/cm^3^. The mass samples and bed conditions are summarised in Table 2. No flame was observed on the surface of the bed. The drops fell centrally into the fluidised bed and they sank into it (due to lower density of bed) and after a while, glycerol samples were burnt inside the fluidised bed. There was a flue gas probe about 15 cm above the surface of the bed, so the flue gases were sampled from the place, where the temperature was above 100 °C. The gas path (to analyzers) was maintained at 170–180 °C; so there was no possibility of any compounds condensation. Quantitative analysis of the ingredients was carried out by infrared absorption spectroscopy (FT-IR, DX-4000, Gasmet Technologies). Registered concentrations are shown in Tables 2–6 and Tables 7–11, respectively for inert and catalytic fluidised bed.

## Conflict of Interest

The authors declare that they have no known competing financial interests or personal relationships which have, or could be perceived to have, influenced the work reported in this article.

## Appendix Supplementary materials

Image, application 1
